# Preventative behaviours and COVID-19 infection in a Canadian cohort of people living with HIV

**DOI:** 10.1186/s12981-023-00571-7

**Published:** 2023-10-20

**Authors:** Keely Hammond, Terry Lee, Branka Vulesevic, Joel Singer, Judy Needham, Ann N. Burchell, Hasina Samji, Sharon Walmsley, Mark Hull, Mohammad-Ali Jenabian, Jean-Pierre Routy, Shari Margolese, Enrico Mandarino, Aslam H. Anis, Curtis L. Cooper, Cecilia T. Costiniuk

**Affiliations:** 1 Department of Medicine and Division of Infectious Diseases and Chronic Viral Illness Service, McGill University Health Centre, Montréal, QC Canada; 2 Canadian Institutes of Health Research (CIHR) Canadian HIV Trials Network, Vancouver, BC Canada; 3 Centre for Health Evaluation and Outcome Sciences, St. Paul’s Hospital, Vancouver, BC Canada; 4 The Ottawa Hospital and Ottawa Hospital Research Institute, Ottawa, ON Canada; 5 School of Population and Public Health, University of British Columbia, Vancouver, Canada; 6 Department of Family and Community Medicine, Faculty of Medicine, University of Toronto, Toronto, ON Canada; 7 MAP Centre for Urban Health Solutions, Li Ka Shing Knowledge Institute, St Michael’s Hospital, Unity Health Toronto, Toronto, ON Canada; 8 British Columbia Centre for Disease Control, Vancouver, BC Canada; 9 Faculty of Health Sciences, Simon Fraser University, Burnaby, BC Canada; 10 Division of Infectious Diseases, Department of Medicine, University of Toronto, Toronto, ON Canada; 11 British Columbia Centre for Excellence in HIV/AIDS, Vancouver, BC Canada; 12 Department of Biological Sciences, Université du Québec à Montréal, Montréal, QC Canada; 13 Infectious Diseases and Immunity in Global Health Research Institute of McGill University Health Centre, 1001 Boulevard Decarie, Room EM2.3226, Montréal, QC H3A 3J1 Canada; 14 Division of Hematology, Department of Medicine, McGill University Health Centre, Montréal, QC Canada; 15 Community Advisory Committee, CIHR Canadian HIV Trials Network, Vancouver, BC Canada; 16 University of Ottawa, Ottawa, ON Canada

**Keywords:** COVID-19 preventative behaviours, Masking, Physical distancing, People living with HIV, COVID-19 immunization

## Abstract

Few studies have examined preventative behaviour practices with respect to COVID-19 among people living with HIV (human immunodeficiency virus). Using a cross-sectional survey from a Canadian Institutes of Health Research Canadian HIV Trials Network study (CTN 328) of people living with HIV on vaccine immunogenicity, we examined the relationships between participant characteristics and behavioural practices intended to prevent COVID-19 infection. Participants living in four Canadian urban centers were enrolled between April 2021–January 2022, at which time they responded to a questionnaire on preventative behaviour practices. Questionnaire and clinical data were combined to explore relationships between preventive behaviours and (1) known COVID-19 infection pre-enrolment, (2) multimorbidity, (3) developing symptomatic COVID-19 infection, and (4) developing symptomatic COVID-19 infection during the Omicron wave. Among 375 participants, 49 had COVID-19 infection pre-enrolment and 88 post-enrolment. The proportion of participants reporting always engaging in preventative behaviours included 87% masking, 79% physical distancing, 70% limiting social gatherings, 65% limiting contact with at-risk individuals, 33% self-isolating due to symptoms, and 26% self-quarantining after possible exposure. Participants with known COVID-19 infection pre-enrolment were more likely to self-quarantine after possible exposure although asymptomatic (65.0% vs 23.4%, p < 0.001; Chi-square test). Participants with multiple comorbidities more likely endorsed physical distancing (85.7% vs 75.5%, p = 0.044; Chi-square test), although this was not significant in logistic regression analysis adjusted for age, sex, race, number of household members, number of bedrooms/bathrooms in the household per person, influenza immunization, and working in close physical proximity to others. Overall, participants reported frequent practice of preventative behaviours.

**Supplementary Information:**

The online version contains supplementary material available at 10.1186/s12981-023-00571-7.

## Introduction

Globally, many people have faced challenges during the COVID-19 pandemic including loss of income and employment, worsened mental health, and decreased access to medical care [1, 2]. The pandemic has also amplified the intersectional vulnerabilities faced by many people living with human immunodeficiency virus (HIV). For example, among people living with HIV in the United States, African–Americans and those with low incomes were more likely to suffer complications following severe COVID-19 infection [3]. People living with HIV may also have difficulty placing trust in the health care system; in one cohort of African–American people living with HIV in the United States, 97% of individuals endorsed at least one COVID-19 mistrust belief and half had COVID-19 vaccine-specific mistrust [4]. By contrast, people living with HIV may be more engaged in COVID-19 preventative behaviours or vaccine uptake than the general population [5, 6]. People living with HIV have known history of activism and high level of community involvement in research. Considering this, more study of COVID-19 preventative behaviours is needed within the population of people living with HIV that can guide new policies and enhance vaccination success.

Since the first global COVID-19 immunization campaign was launched, attitudes and uptake of the COVID-19 vaccine in people living with HIV have been much more extensively researched than behavioural practices (e.g. mask-wearing, avoiding large gatherings). In this study, we sought to understand the relationships between preventative behaviours and COVID-19 infection in a multi-centre, cross-sectional study of people living with HIV in Canada. We addressed this topic through four questions:

1. Does previous known COVID-19 infection influence preventative behaviours among PLWH?
2. Is participant multimorbidity (presence of multiple comorbidities) associated with preventative behaviour practices among PLWH?
3. Are preventative behaviour practices, living in a crowded space, and working in close proximity to others associated with COVID-19 transmission among PLWH?
4. Are preventative behaviour practices and/or uptake of COVID-19 vaccination associated with developing symptomatic COVID-19 infection during the highly contagious Omicron wave among PLWH?

## Methods

Our study population comprised people living with HIV living in Montréal, Ottawa, Toronto and Vancouver, Canada aged at least 16 years who had received no more than two COVID-19 immunizations at the time of enrolment. The immunization requirement was part of the inclusion criteria for a separate study on COVID-19 vaccine immunogenicity [7]. Participants were engaged in HIV care and were recruited through participating medical clinics. Participants were enrolled between April 2021 and January 2022. At enrolment, demographic data were collected, together with the COVID-19 Immunity Task Force (CITF) Standardized Core Survey Data Elements questionnaire [8] [Additional file 1]. This cross-sectional questionnaire captured self-reported preventative behaviours including masking, physical distancing, avoiding crowds, limiting physical greetings (hugs and handshakes), avoiding visits with vulnerable individuals, self-isolating if sick, and self-quarantining if suspected exposure. Frequency of behaviours was ranked on a five-point scale from ‘never’ to ‘always’, with only those reporting ‘always’ included as engaging in a specific behaviour. HIV viral load, CD4 cell count was obtained from samples taken within 12 months of enrolment. COVID-19-specific antibody testing was performed from samples obtained at enrolment and at subsequent visits within the study period. Participant comorbidities including obesity, cardiac disease, lung disease, dyslipidemia, diabetes, and other significant comorbidities were also recorded at the time of enrolment using the patient questionnaire and chart review.

From the total study population, responses from appropriate subsets of participants were analyzed to address each of the four aforementioned questions, as described in Table 1. Statistical analysis was performed to assess for significant differences between demographics and CITF questionnaire responses with t-test, chi-square test, and Fisher exact test used as appropriate. Multivariable logistic regression was performed to assess for associations between outcomes and predictors of interest while accounting for other factors that might confound the association based on prior knowledge. No imputation was performed to impute the missing data as this is mainly a descriptive study. Conduct of this study was approved by the Canadian Institutes of Health Research Canadian HIV Trials Network (CTN) Scientific Review Committee and Community Advisory Committee, as well as by each site’s Research Ethics Board as previously outlined [7].

**Table 1:** Demographic characteristics, immunization status, and preventative behaviour practices among a cohort of people living with HIV in Canada; the total cohort (n = 375) is separated into four overlapping groups with the aim of answering four questions as listed in footnote 1

Characteristics	Does previous known COVID-19 infection influence preventative behaviours?(n = 351)	Is participant multimorbidity associated with preventative behaviours?^1^ (n = 341)	Are preventative behaviours, living in a crowded space, and working in close proximity to others associated with COVID-19 infection?^1^ (n = 326)	Are preventative behaviours and/or uptake of vaccination associated with symptomatic infection during Omicron?^1^ (n = 283)
Age in years, mean (SD)	52.0 (13.5)	51.9 (13.4)	52.0 (13.6)	52.3 (13.5)
Current sex, n (%)				
Male	260 (74.1)	257 (75.4)	245 (75.2)	216 (76.3)
Female	89 (25.4)	82 (24.0)	79 (24.2)	65 (23.0)
Prefer to self-describe	2 (0.6)	2 (0.6)	2 (0.6)	2 (0.7)
Self-declared race or ethnicity, n (%)				
White	196 (56.5)	197 (58.5)	193 (59.8)	172 (61.2)
Black	71 (20.5)	66 (19.6)	59 (18.3)	48 (17.1)
Asian	31 (8.8)	29 (8.5)	27 (8.3)	22 (7.8)
Latin American	27 (7.7)	24 (7.0)	24 (7.4)	21 (7.4)
Indigenous	6 (1.7)	6 (1.8)	5 (1.5)	5 (1.8)
Other	19 (5.4)	18 (5.3)	18 (5.5)	16 (5.7)
No response	4 (1.1)	4 (1.2)	3 (0.9)	2 (0.7)
Time of enrolment based on COVID-19 waves, n (%)				
April–May 2021 (Alpha)	89 (25.4)	83 (24.3)	85 (26.1)	73 (25.8)
June–July 2021 (Alpha/Delta)	109 (31.1)	115 (33.7)	104 (31.9)	88 (31.1)
August–September 2021 (Delta)	82 (23.4)	74 (21.7)	73 (22.4)	65 (23.0)
October–November 2021 (Delta)	53 (15.1)	50 (14.7)	48 (14.7)	43 (15.2)
December 2021–January 2022 (Omicron BA.1)	18 (5.1)	19 (5.6)	16 (4.9)	14 (4.9)
Duration of HIV infection in years, median (IQR)	16.0 (7.0–24.0)	16.0 (7.0–25.0)	16.0 (7.0–25.0)	17.0 (7.0–24.5)
HIV controlled and stable^2^, n/total n available (%)	169/326 (51.8)	171/323 (52.9)	161/301 (53.5)	143/263 (54.4)
Immune non-responders^3^, n/total n available (%)	29/331 (8.8)	29/322 (9.0)	27/307 (8.8)	23/267 (8.6)
Comorbidities				
Two or more comorbidities, n/total n available (%)	97/344 (28.2)	95/341 (27.9)	89/319 (27.9)	76/277 (27.4)
Obesity, n/total n available (%)	74/331 (22.4)	74/325 (22.8)	66/307 (21.5)	54/268 (20.1)
Cardiac disease (including hypertension), n (%)	66 (18.8)	62 (18.1)	60 (18.4)	54 (19.0)
Dyslipidemia, n (%)	47 (13.6)	45 (13.2)	43 (13.4)	40 (14.3)
Chronic lung disease, n (%)	30 (8.7)	30 (8.8)	27 (8.4)	23 (8.2)
Diabetes, n (%)	26 (7.5)	26 (7.6)	24 (7.5)	23 (8.2)
Other, n (%)	105 (29.9)	102 (29.9)	97 (29.7)	83 (29.3)
Highest level of education, n (%)				
Less than or including secondary school/trade certificate, apprenticeship, non-university certificate or diploma from a community college, CEGEP	212 (63.5)	208 (64.0)	196 (63.0)	168 (62.2)
University undergraduate degree or graduate degree	122 (36.5)	117 (36.0)	115 (37.0)	102 (37.8)
Prefer not to answer/no response	17	16	15	13
COVID-19 infection prior to enrolment, n (%)				
Self-reported	25 (7.1)	n/a	n/a	n/a
By antibody testing only	n/a	24 (7.0)	n/a	n/a
COVID-19 infection after enrolment, n (%)				
Self-reported	54 (15.4)	54 (15.8)	54 (16.6)	51 (18.0)
By antibody testing only	34 (9.7)	34 (10.0)	34 (10.4)	n/a
Sought testing for COVID-19 infection prior to enrolment, n/total n available (%)	120/342 (35.1)	98/332 (29.5)	95/317 (30.0)	87/276 (31.5)
Number of COVID-19 vaccine doses received prior to study enrolment, n (%)				
None	95 (27.1)	88 (25.8)	87 (26.7)	69 (24.4)
Received 1 dose of a 2-dose schedule	130 (37.0)	130 (38.1)	121 (37.1)	105 (37.1)
Received 2 doses of a 2-dose schedule, or 1 dose of a 1-dose schedule	126 (35.9)	123 (36.1)	118 (36.2)	109 (38.5)
At least 3 doses of COVID-19 vaccine received by Dec. 1, 2021, n (%)	58 (16.5)	55 (16.1)	56 (17.2)	52 (18.4)
At least 4 doses of COVID-19 vaccine received by Sept. 1, 2022, n (%)	76 (21.7)	73 (21.4)	74 (22.7)	66 (23.3)
Participants reporting usually getting influenza immunization, n/total n available (%)	259/341 (76.0)	251/332 (75.6)	241/317 (76.0)	215/276 (77.9)
Number of individuals (including participant) living in household, n (%)				
Unknown	15	16	15	11
One	163 (48.5)	158 (48.6)	153 (49.2)	132 (48.5)
Two or more	173 (51.5)	167 (51.4)	158 (50.8)	140 (51.5)
At least one bedroom in household per person, n/total n available (%)	267/330 (80.9)	261/319 (81.8)	251/305 (82.3)	219/267 (82.0)
At least one bathroom in household per person, n/total n available (%)	214/328 (65.2)	212/317 (66.9)	204/303 (67.3)	177/265 (66.8)
Traveled outside of home province since January 2020, n/total n available (%)	82/342 (24.0)	79/331 (23.9)	77/317 (24.3)	69/275 (25.1)
Paid or unpaid work in close physical proximity to other people, n/total n available (%)	109/339 (32.2)	103/327 (31.5)	100/316 (31.6)	92/276 (33.3)
Participants attending at least one gathering of ten or more since March 2020, n/total n available (%)	130/318 (40.9)	126/309 (40.8)	120/298 (40.3)	110/262 (42.0)

	Does previous known COVID-19 infection influence preventative behaviours?^4^		Is participant multimorbidity associated with preventative behaviours?^5^		Are preventative behaviours, living in a crowded space, and working in close proximity to others associated with COVID-19 infection?^6^		Are preventative behaviours and/or uptake of vaccination associated with symptomatic infection during Omicron?^7^	
Answer to numbered question	No (n = 326)	Yes (n = 25)	No (n = 246)	Yes (n = 95)	No (n = 238)	Yes (n = 88)	No (n = 232)	Yes (n = 51)
Preventative behaviours, % practising behaviours								
Wearing a mask in public places	86.8 (277/319)	84 (21/25)	86.4 (209/242)	92.3 (84/91)	86.1 (199/231)	88.6 (78/88)	85.8 (194/226)	90.2 (46/51)
Practicing physical distancing in public places	78.3 (249/318)	84 (21/25)	75.5 (182/241)	85.7 (78/91)*	79.1 (182/230)	76.1 (67/88)	79.1 (178/225)	76.5 (39/51)
Avoiding crowded places/gatherings	70.3 (233/317)	60 (15/25)	67.9 (163/240)	76.9 (70/91)	72.1 (165/229)	65.9 (58/88)	71.4 (160/224)	64.7 (33/51)
Avoiding common greetings	68.3 (218/319)	76 (19/25)	65.3 (158/242)	75.8 (69/91)	71.0 (164/231)	61.4 (54/88)	70.8 (160/226)	62.7 (32/51)
Limiting contact with people at higher risk	64.5 (187/290)	69.6 (16/23)	65 (141/217)	63.5 (54/85)	64.0 (135/211)	65.8 (52/79)	64.1 (132/206)	63.8 (30/47)
Self-isolating because the participant thought infected with COVID-19	32.5 (82/252)	56.5 (13/23)*	33.7 (64/190)	26.8 (19/71)	27.9 (50/179)	43.8 (32/73)*	27.3 (48/176)	50.0 (22/44)*
Self-quarantining because the participant thought exposed to COVID-19 but showed no symptoms	23.4 (60/256)	65.0 (13/20)*	26.6 (51/192)	18.1 (13/72)	20.3 (37/182)	31.1 (23/74)	(35/179)	(13/44)

^1^The four questions are as follows: (1) Does previous known COVID-19 infection (excluding those with positive antibody testing but no knowledge of prior infection) influence preventative behaviours?, (2) Is participant multimorbidity associated with preventative behaviour practices?, (3) Are preventative behaviour practices, living in a crowded space, and working in close proximity to others associated with COVID-19 infection?, and (4) Are preventative behaviour practices and/or uptake of COVID-19 vaccination associated with developing symptomatic COVID-19 infection during the highly contagious Omicron wave?

^2^HIV controlled and stable defined by CD4 cell count ≥ 350, suppressed HIV viral load, and ≤ 1 comorbidity

^3^Immune non-responders defined by CD4 < 350 and CD4/CD8 < 0.75 despite suppressed HIV viral load

^4^Effect of previous known COVID-19 infection on preventative behaviours: 24 were excluded from the analysis as they reported not having COVID-19 infection prior to study enrolment but had positive antibody testing

^5^Effect of multimorbidity on preventative behaviours: 25 were excluded from the analysis for this question as they had self-reported COVID-19 infection prior to enrolment; we hypothesized this would affect their decision-making regarding preventative behaviours

^6^Preventative behaviours and COVID-19 infection risk: 49 were excluded from the analysis for this question as they had either self-reported COVID-19 infection prior to enrolment or positive antibody testing at the time of enrolment

^7^Preventative behaviours and Omicron infection risk: 92 were excluded from the analysis; 57 had COVID-19 infection before study enrolment or before the Omicron wave, 29 had asymptomatic COVID-19 during the Omicron wave (positive antibody testing but did not self-report infection), and 6 dropped out from the study prior to the Omicron wave

^*^ denotes p ≤ 0.05

## Results 

Among the 375 participants enrolled in the study, the mean age was 52.0 years (standard deviation 13.3 years) with a range of 19.7–83.5 years. Detailed characteristics of participants are presented in Table 1. The median duration of HIV infection was 17.0 years with interquartile range 7.0–24.0 years. The overall proportion of participants with well-controlled HIV infection (defined as CD4 ≥ 350 cells/mm^3^, suppressed HIV viral load), and low number of comorbidities (one or fewer) was 52%. One-third of participants (28.1%) had two or more comorbidities. Most participants (69.1%) had achieved more than a secondary school education (secondary school education comprises schooling up to age 17–18 in Canada). A total of 49 participants had documented COVID-19 infection before study enrolment based on either self-report (n = 25, 51%) or laboratory testing (n = 24, 49%). Eighty-eight participants contracted COVID-19 infection during the study period, up until April 2022, based on either of self-report (n = 54, 61%) and laboratory testing (n = 34, 39%). Of note, the vaccine immunogenicity study is still ongoing so the total number of COVID-19 infections during the total study period is currently unknown. Overall, preventative behaviours were frequently practiced in the cohort, with 87% masking in public, 79% distancing, 70% avoiding large gatherings, and 65% limiting contact with vulnerable persons.

### Does previous known COVID-19 infection influence preventative behaviours?

**Fig. 1:**
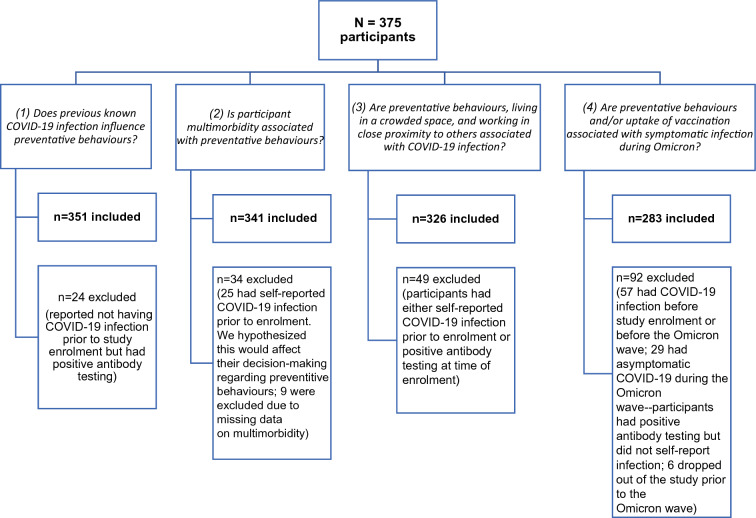
Flowchart demonstrating subsets of the cohort used to answer four questions related to participant demographics and COVID-19 preventative behaviours

To address this question, we excluded individuals with positive serum COVID-19 antibody testing (presumed prior infection) but no knowledge of prior infection (n = 24). A detailed explanation of the participant subsets used in each of the four questions is found in Fig. 1. Participants reporting prior known COVID-19 infection (based on self-report) (n = 25) were more likely to identify as non-white (p < 0.001), less likely to have stable HIV infection (32.0% vs 53.5%, p = 0.039), have more household members (p < 0.001), fewer household bedrooms and bathrooms per person (p = 0.021 and p = 0.006, respectively), and were more likely to be employed in health care (p < 0.001) than those not reporting prior infection. There were no significant differences in the other demographic factors between the prior known infection and non-infected groups. In response to the preventative behaviours survey, participants with prior known COVID-19 were more likely to self-quarantine when thought to have been exposed to COVID-19 but were not symptomatic (p < 0.001) and self-isolate when thought to been infected with COVID-19 (p = 0.021). These differences remained statistically significant after adjusting for age, sex and the aforementioned patient characteristics that were different between groups (aOR = 6.72 [95% CI: 1.98, 22.84], p = 0.002 and 3.50 [95% CI: 1.09, 11.21], p = 0.035 respectively). These were the only significant differences in preventative behaviours between groups.

### Is participant multimorbidity associated with preventative behaviour practices?

Participants in the multimorbidity group were more likely to be older (mean age 59.2 years vs 49.0 years, p < 0.001), live in a household with fewer members (p = 0.020), have more bedrooms and bathrooms in the household per person (p = 0.019 and p = 0.008, respectively), usually get an influenza immunization (p = 0.045), and less likely to be performing paid or unpaid work in close physical proximity to others (p = 0.035). They were more likely to be vaccinated with four doses against COVID-19 by September 2022 (33.7% vs 16.7%, p = 0.001). In response to the preventative behaviours survey, participants in the multimorbidity group were more likely to be practicing physical distancing (85.7% vs 75.5%, p = 0.044). This difference, however, was no longer statistically significant after adjusting for participant characteristics (age, sex, race, number of household members, number of bedrooms and bathrooms in the household per person, uptake of influenza immunization and performing paid or unpaid work in close physical proximity to others) (aOR = 1.74 [95% CI: 0.84, 3.58], p = 0.140), and no other significant differences in preventative behaviours between groups were noted.

### Are preventative behaviour practices, living in a crowded space, and working in close proximity to others associated with COVID-19 infection?

The participants in the COVID-19 infection group were more likely to have fewer bedrooms per person (mean 1.0 vs 1.3, p = 0.006). There were no identified differences in the proportion of participants performing paid or unpaid work in close physical proximity to others between those with and without COVID-19 infection (28.2% vs 32.9%, p = 0.429). There were no identified differences in preventative behavior practices between those with and without baseline COVID-19 infection.

### Are preventative behaviour practices and/or uptake of COVID-19 vaccination associated with developing symptomatic COVID-19 infection during the highly contagious Omicron wave?

In Canada, the Omicron wave began in late November 2021 [9]. Participants in the Omicron infection group were more likely to have been tested for COVID-19 at some point before study enrolment (p = 0.015). There was no statistical difference in Omicron infection rate by COVID-19 vaccination status at the start of the Omicron wave (26.9% versus 16.0% for those who received 3 vaccine doses versus less than 3 doses, p = 0.065). The finding was the same after adjusting for age, sex, race, multimorbidity, number of household members, number of bedrooms and bathrooms in the household per person and performing paid/unpaid work in close physical proximity to others (aOR = 1.84 [95% CI: 0.80, 4.22], p = 0.150). There were no significant differences in preventative behaviours between those sustaining COVID-19 infection during the Omicron wave and those not infected during this time period.

## Discussion

Using data from our cohort of people living with HIV, we examined four questions regarding COVID-19 preventative behaviours. Another study done in the Canada in general population assessed determinants of adherence to major coronavirus preventive behaviours, including demographics, attitudes and concerns and showed that adherence to COVID-19 prevention behaviours was worse among men, younger adults, and workers, and deteriorated over time [10]. We did not observe these differences. Among those having prior known infection with COVID-19, the only difference noted in preventative behaviours was an increased likelihood of self-quarantining after a suspected exposure. Participants engaged in work with close physical proximity to others did not report different preventative behaviours or COVID-19 infection proportions. Multimorbidity was associated with more physical distancing, although there were also multiple demographic factors noted to be different in this group (increased vaccine uptake and less crowding at home and work). In the highly contagious Omicron wave, we did not observe any differences in vaccine uptake or preventative behaviours between those who did and did not sustain infection. Overall, preventative behaviours were practiced in a high proportion of the cohort, with 87% masking in public, 79% distancing, 70% avoiding large gatherings, and 65% limiting contact with vulnerable persons. In a 2020 Canadian survey cohort of the general population, over 70% always reported masking in public and staying home when sick while over 50% avoided large gatherings; only 40% engaged in physical distancing [11].

Preventative behaviours including masking, physical distancing, and limiting gatherings have had high uptake globally in people living with HIV. In a South African cohort of people living with HIV, 80% changed one or more activities based on public health recommendations [12]. One United States cohort of 149 people living with HIV reported engaging in an average of 2.8 (SD 1.4, range 0–5) physical distancing behaviours [13]. In a cohort of 545 primarily male Indonesian people living with HIV, 70% reported practicing preventative behaviours [2]. Among 376 Rwandan people living with HIV, factors associated with the increased practice of preventative behaviours included duration of antiretroviral therapy and female gender [14]. Increasing age had a consistent association with preventative behaviours in one rapid review of the general population (not specific to those living with HIV) in developed countries, while health status and education did not show consistent effects [15].

Limited data exist on the effects of prior COVID-19 infection on preventative behaviours or on the influence of working in close physical proximity to others on COVID-19 behaviours in people living with HIV. Greater vaccine uptake among those with multimorbidity and/or older age has been reported in a South African cohort of people living with HIV [12]. In contrast, a Chinese cohort of people living with HIV was less likely to receive COVID-19 vaccination [16]. Fear of disclosure of HIV status at vaccination appointments was reported in this later assessment which may explain the heterogeneity of findings across reports.

We observed no difference in vaccination status between participants sustaining Omicron infection and those not infected. We note that studies in the general population have shown a less protective effect of original vaccine formulations against the Omicron variants although behavioural differences during the Omicron wave may have also played a role in our cohort [17].

Our study has several limitations: the entire cohort was participating in COVID-19 vaccination programs to some degree and had easy access to provincial and federal public health programs for testing and education. This may limit the generalization of our results to settings where public health infrastructure is not available to disseminate information and vaccines. It also encapsulates behaviours for only a portion of people living with HIV who consented to vaccination. Data was collected only at the beginning of Omicron wave resulting in small number of participants being infected by COVID-19 Omicron variant and therefore may not be fully generalizable to Omicron and subsequent waves.

In summary, our Canadian cohort of people living with HIV reported high rates of preventative behaviour practices. We found differences in preventative behaviours among those with prior COVID-19 infection and in those with multimorbidity suggesting these are key motivating factors in facilitating preventative behaviours.

### Supplementary Information

**Additional file 1. **HIV-COV CITF CDE baseline questionnaire.

## Data Availability

The datasets used and analyzed during the current study are available from the corresponding author on reasonable request.

## Abbreviations

CIHR: Canadian Institutes of Health Research
CTN: Canadian HIV Trials Network
HIV: Human immunodeficiency virus
