# Supplementary material for: Preventative behaviours and COVID-19 infection in a Canadian cohort of people living with HIV
Source: AIDS Res Ther. 2023 Oct 20;20:73. doi: 10.1186/s12981-023-00571-7 (PMC10588118; doi:10.1186/s12981-023-00571-7)
Supplement: Supplementary file 1 — Additional file 1. HIV-COV CITF CDE baseline questionnaire. [file 12981_2023_571_MOESM1_ESM.pdf]

CTN 328: HIV-COV

CITF CDE BASELINE QUESTIONNAIRE

This version is only administered at Screening

Please answer all questions unless otherwise indicated

Participant ID: ___ ___ ___ ___ ___ ___

Section 1: Demographics

1. Date (DD-MMM-YYYY):

   ___ ___ / ___ ___ / ___ ___ ___ ___

2. What is your age?

   ___ ___ ___ YRS ___ ___ MO OR ☐ Prefer not to answer

3. What was your assigned sex at birth?

   ◯ Male
   ◯ Female
   ◯ Prefer to self-describe (specify) ______________________________
   ◯ Prefer not to answer

4. What is your sex now?

   ◯ Male
   ◯ Female
   ◯ Prefer to self-describe (specify) ______________________________
   ◯ Prefer not to answer

5. What is your gender (how do you currently self-identify)?

- ◯ Male
- ◯ Female
- ◯ Non-binary, genderqueer, agender or a similar identity
- ◯ Two-spirit
- ◯ Prefer to self-describe (specify) ______________________________
- ◯ Prefer not to answer

6. Are you an Indigenous person originating from North America?

If NO or Prefer not to answer, please proceed to Q9

- ◯ No
- ◯ Yes
- ◯ Prefer not to answer

7. Which of the following groups do you belong to? Please select all that apply.

Only answer if Q6 = YES

- ☐ First Nations
- ☐ Inuit
- ☐ Metis
- ☐ Non-status First Nations
- ☐ Other Indigenous (specify) ______________________________
- ☐ Prefer not to answer

8. Do you live on reserve?

Only answer if Q7 = First Nations

- ◯ Yes
- ◯ No
- ◯ Prefer not to answer

9. How would you describe your ethnicity or race? Please select all that apply.

If you are an Indigenous person and answered YES to Q6, select any other that apply.

- ☐ White
- ☐ South Asian
- ☐ Chinese
- ☐ Black
- ☐ Filipino
- ☐ Latin American
- ☐ Arab
- ☐ Southeast Asian
- ☐ West Asian
- ☐ Korean
- ☐ Japanese
- ☐ Prefer to self-describe (specify) ______________________________
- ☐ Prefer not to answer

10. What are the first three digits of your postal code?

___ ___ ___ OR ☐ Prefer not to answer

11. What is the highest level of education you have completed?

◯ Less than high school graduation
◯ High school graduation
◯ Trade certificate, vocational school, or apprenticeship training
◯ Non-university certificate or diploma from a community college, CEGEP
◯ University Bachelor's degree
◯ University graduate degree (Master's, Doctorate, etc.)
◯ Prefer not to answer

12. How many people live in your household, including yourself?

________ OR ☐ Prefer not to answer

13. How many bedrooms are in your household?

________ OR ☐ Prefer not to answer

14. How many bathrooms are in your household?

________ OR ☐ Prefer not to answer

Section 2: COVID-19

15. Do you think you have had COVID-19?

If NO or Prefer not to answer, please proceed to Q18

◯ No
◯ Yes
◯ Prefer not to answer

16. Why do you think you have had COVID-19? Please select all that apply.

Only answer if Q15 = YES

☐ Symptom review online
☐ Symptom profile
☐ Nasal/throat test result
☐ Health care provider
☐ Contact with case
☐ Other (specify) ______________________________
☐ Prefer not to answer

17. Were you hospitalized due to COVID-19?

Only answer if Q15 = YES

○ No
○ Yes
○ Prefer not to answer

18. Have you ever been tested for an active COVID-19 infection (using nasopharyngeal/throat swab, saliva, or gargle test)?

If NO or Prefer not to answer, please proceed to Q21

○ No
○ Yes
○ Prefer not to answer

19. If yes, how may times have you been tested?

Only answer if Q18 = YES

_________ OR ☐ Prefer not to answer

20.1 Answer the following questions about the **first COVID-19 test**, if applicable.

20.1.a What was the date of the **first** test?

___ ___ DD / ___ ___ MO /___ ___ ___ ___ YR

20.1.b What was the result of the **first** test?

○ Negative
○ Positive
○ Don't know

20.1.c Did you have any symptoms of COVID when you had this test?

- ◯ No
- ◯ Yes
- ◯ Don't know

20.1.d If yes, what symptoms did you have?

Only answer if Q20.1.c = YES

- ☐ Cough
- ☐ Fever
- ☐ Shortness of breath
- ☐ Sore muscles
- ☐ Headache
- ☐ Sore throat
- ☐ Diarrhea
- ☐ Decreased sense of smell or taste
- ☐ Other (specify) ______________________________

20.2 Answer the following questions about the **second COVID-19 test**, if applicable.

20.2.a What was the date of the **second** test?

___ ___ DD / ___ ___ MO /___ ___ ___ ___ YR

20.2.b What was the result of the **second** test?

- ◯ Negative
- ◯ Positive
- ◯ Don't know

20.2.c Did you have any symptoms of COVID when you had this test?

- ◯ No
- ◯ Yes
- ◯ Don't know

20.2.d If yes, what symptoms did you have?

Only answer if Q20.2.c = YES

- ☐ Cough
- ☐ Fever
- ☐ Shortness of breath
- ☐ Sore muscles
- ☐ Headache
- ☐ Sore throat
- ☐ Diarrhea
- ☐ Decreased sense of smell or taste
- ☐ Other (specify) ______________________________

20.3 Answer the following questions about the **third COVID-19 test**, if applicable.

20.3.a What was the date of the **third** test?

___ ___ DD / ___ ___ MO /___ ___ ___ ___ YR

20.3.b What was the result of the **third** test?

- ○ Negative
- ○ Positive
- ○ Don't know

20.3.c Did you have any symptoms of COVID when you had this test?

- ○ No
- ○ Yes
- ○ Don't know

20.3.d If yes, what symptoms did you have?

Only answer if Q20.3.c = YES

- ☐ Cough
- ☐ Fever
- ☐ Shortness of breath
- ☐ Sore muscles
- ☐ Headache
- ☐ Sore throat
- ☐ Diarrhea
- ☐ Decreased sense of smell or taste
- ☐ Other (specify) ______________________________

20.4.a Have you **tested positive** for COVID-19 (using nasopharyngeal, throat swab, saliva or gargle test) on a test that wasn't included the questions above (that is, on the **4th or later test**)?

If NO, please proceed to Q21

- ○ No
- ○ Yes

20.4.b If yes, what was the date the first time you tested positive?

Only answer if Q20.4.a = YES

___ ___ DD / ___ ___ MO ___ ___ ___ ___ YR

Section 3: Exposure

21.a Have you traveled outside of your home province since **January 2020**?

If NO, please proceed to Q23

- ○ No
- ○ Yes
- ○ Prefer not to answer

21.b If you think you had COVID, did you travel in the 6 months before your symptoms began?

Only answer if Q15 = YES

- ○ No
- ○ Yes
- ○ Prefer not to answer

22. What province(s)/territory(ies) or country(ies) did you travel to? Select all that apply.

Only answer if Q21.a or Q21.b = YES. Otherwise, please proceed to Q23.

- ☐ Alberta
- ☐ British Columbia
- ☐ Manitoba
- ☐ New Brunswick
- ☐ Newfoundland and Labrador
- ☐ Northwest Territories
- ☐ Nova Scotia
- ☐ Nunavut
- ☐ Ontario
- ☐ Prince Edward Island
- ☐ Quebec
- ☐ Saskatchewan
- ☐ Yukon

OR ☐ Prefer not to answer

List countries you travelled to (separated by a comma):

____

____

____

____

____

23.a Do you do either paid or unpaid work in an environment where you work in close proximity to other people?

If NO or Prefer not to answer, please proceed to Q24

- ○ No
- ○ Yes
- ○ Prefer not to answer

23.b If yes, have you been working in any of the following occupations or worksites in the past year? Please select all that apply.

Only answer if Q23.a = YES

- ☐ Hospital or health care facility
- ☐ First responder (paramedic/firefighter/police officer)
- ☐ Childcare worker
- ☐ Correctional officer
- ☐ Teacher or other school staff
- ☐ Transit driver
- ☐ Food service industry
- ☐ Grocery store
- ☐ Pharmacy
- ☐ Hairdresser or barber
- ☐ Aesthetician
- ☐ Flight attendant
- ☐ Factor worker
- ☐ Other (specify)

____

- ☐ Prefer not to answer

24.a How many times have you been in a gathering of 10 or more since **March 2020**?

________ OR ☐ Prefer not to answer

24.b If you think you have had COVID, how many times were you in gatherings of more than 10 people in the 6 months before your symptoms began?

Only answer if Q15 = YES

________ OR ☐ Prefer not to answer

Section 4: Health and Health Behaviours

25. Do you currently smoke tobacco?

- ○ No
- ○ Yes
- ○ Prefer not to answer

26. If yes, how often do you smoke tobacco?

Only answer if Q25 = YES

- ○ Less than daily
- ○ Daily

27. Do you currently use e-cigarettes (vape)?

- ○ No
- ○ Yes
- ○ Prefer not to answer

28. If yes, how often do you use e-cigarettes (vape)?

Only answer if Q27 = YES

- ○ Less than daily
- ○ Daily

29. Have you been diagnosed by a physician with any of the following chronic medical conditions? Please provide an answer for each condition.

		**Yes**	**No**	**Don't Know**	**Prefer not to answer**
a.	Hypertension				
b.	Diabetes				
c.	Asthma				
d.	Chronic Lung Disease				
e.	Chronic Heart Disease				
f.	Chronic Kidney Disease				
g.	Liver Disease				
h.	Cancer				
i.	Chronic Blood Disorder				
j.	Immune Suppressed				
k.	Chronic Neurological Disorder				

30. What is your current weight (circle units)?

________ kg / lbs OR ☐ Prefer not to answer

31. What is your current height?

____ . ____ ____ m OR ____ ft ____ ____ in OR ☐ Prefer not to answer

32. Do you have a family physician/primary care provider?

○ No
○ Yes
○ Don't know
○ Prefer not to answer

33. Do you usually get a flu shot?

○ No
○ Yes
○ Prefer not to answer

34. Indicate if, or how often you have done the following since **March 2020**?

		Never	Rarely	Occasionally	Often	Always	Prefer not to answer
a.	Worn a mask in public places						
b.	Practiced physical distancing in public places						
c.	Avoided crowded places/gatherings						
d.	Avoided common greetings (such as a handshake or hug)						

		Never	Rarely	Occasionally	Often	Always	N/A	Prefer not to answer
e.	Limited contact with people at higher risk (e.g., an elderly relative)							

		No	Yes	N/A	Prefer not to answer
f.	Self-isolated because you thought you were infected with COVID-19				
g.	Self-quarantined because you may have been exposed to COVID-19, but did not show symptoms				

35. **If you think you have had COVID**, have you done the following in the **6 months before your symptoms began**? (indicate how often).

Only answer if Q15 = YES

		**Never**	**Rarely**	**Occasion ally**	**Often**	**Always**	**N/A**	**Prefer not to answer**
a.	Worn a mask in public places							
b.	Practiced physical distancing in public places							
c.	Avoided crowded places/gatherings							
d.	Avoided common greetings (such as handshake or hug)							
e	Limited contact with people at higher risk (e.g., an elderly relative)							

		**No**	**Yes**	**N/A**	**Prefer not to answer**
f.	Self-isolated because you thought you were infected with COVID-19				
g.	Self-quarantined because you may have been exposed to COVID-19, but did not show symptoms				

Section 5: Vaccine

36. Have you been vaccinated against COVID-19? Answer YES if you have received at least one dose of the COVID-19 vaccine.

If NO or Prefer not to answer, proceed to the end of this questionnaire

- ○ No
- ○ Yes
- ○ Prefer not to answer

37. How many doses of the COVID-19 vaccine have you received so far?

Only answer if Q36 = YES

- ○ One
- ○ Two
- ○ More than two

38. When did you receive the **first dose** of the COVID-19 vaccine?

Only answer if Q36 = YES

___ ___ DD / ___ ___ MM / ___ ___ ___ ___ YR

39. Which vaccine did you receive for the **first dose**?

Only answer if Q36 = YES

- ○ Pfizer and BioNTech mRNA vaccine
- ○ Moderna mRNA vaccine
- ○ AstraZeneca Oxford vaccine
- ○ Other (specify) ______________________________
- ○ Janssen (Johnson & Johnson) vaccine
- ○ Don't know
- ○ Prefer not to answer

40. Were you pregnant when you received the **first dose**?

Only answer if Q36 = YES

- ○ No
- ○ Yes
- ○ N/A

41. If yes, what trimester were you in when you received the **first dose**?

Only answer if Q40 =YES

- ○ First
- ○ Second
- ○ Third

42. When did you receive the **second dose** of the COVID-19 vaccine?

Only answer if Q37 = TWO or MORE THAN TWO

___ ___ DD / ___ ___ MM / ___ ___ ___ ___ YR

43. Which vaccine did you receive for the **second dose**?

Only answer if Q37 = TWO or MORE THAN TWO

- ◯ Pfizer and BioNTech mRNA vaccine
- ◯ Moderna mRNA vaccine
- ◯ AstraZeneca Oxford vaccine
- ◯ Other (specify) ______________________________
- ◯ Janssen (Johnson & Johnson) vaccine
- ◯ Don't know
- ◯ Prefer not to answer

44. Were you pregnant when you received the **second dose**?

Only answer if Q37 = TWO or MORE THAN TWO

- ◯ No
- ◯ Yes
- ◯ N/A

45. If yes, what trimester were you in when you received the **second dose**?

Only answer if Q44 =YES

- ◯ First
- ◯ Second
- ◯ Third

END OF QUESTIONNAIRE
